# Emerging issues in paediatric health research consent forms in Canada: working towards best practices

**DOI:** 10.1186/1472-6939-14-5

**Published:** 2013-01-30

**Authors:** Edward S Dove, Denise Avard, Lee Black, Bartha M Knoppers

**Affiliations:** 1 Department of Human Genetics, Centre of Genomics and Policy, Faculty of Medicine, McGill University, 740 Avenue Dr. Penfield, Suite 5200, Montreal, QC, H3A 0G1, Canada

**Keywords:** Children, Confidentiality, Consent, ELSI, Paediatric research, Research ethics, Return of results, Withdrawal

## Abstract

**Background:**

Obtaining a research participant’s voluntary and informed consent is the bedrock of sound ethics practice. Greater inclusion of children in research has led to questions about how paediatric consent operates in practice to accord with current and emerging legal and socio-ethical issues, norms, and requirements.

**Methods:**

Employing a qualitative thematic content analysis, we examined paediatric consent forms from major academic centres and public organisations across Canada dated from 2008–2011, which were purposively selected to reflect different types of research ethics boards, participants, and studies. The studies included biobanking, longitudinal studies, and gene-environment studies. Our purpose was to explore the following six emerging issues: (1) whether the scope of parental consent allows for a child’s assent, dissent, or future consent; (2) whether the concepts of risk and benefit incorporate the child’s psychological and social perspective; (3) whether a child’s ability to withdraw is respected and to what extent withdrawal is permitted; (4) whether the return of research results includes individual results and/or incidental findings and the processes involved therein; (5) whether privacy and confidentiality concerns adequately address the child’s perspective and whether standard data and/or sample identifiability nomenclature is used; and (6) whether retention of and access to paediatric biological samples and associated medical data are addressed.

**Results:**

The review suggests gaps and variability in the consent forms with respect to addressing each of the six issues. Many forms did not discuss the possibility of returning research results, be they individual or general/aggregate results. Forms were also divided in terms of the scope of parental consent (specific versus broad), and none discussed a process for resolving disputes that can arise when either the parents or the child wishes to withdraw from the study.

**Conclusions:**

The analysis provides valuable insight and evidence into how consent forms address current ethical issues. While we do not thoroughly explore the contexts and reasons behind consent form gaps and variability, we do advocate and formulate the development of best practices for drafting paediatric health research consent forms. This can greatly ameliorate current gaps and facilitate harmonised and yet contextualised approaches to paediatric health research ethics.

## Background

Obtaining a research participant’s voluntary and informed consent is the bedrock of sound legal and ethics practice. From an ethical and human rights perspective, informed consent protects the research participant from potential harm and promotes his or her autonomy and dignity. From a legal perspective, it can act as a waiver to the common law tort of battery or negligence in medical research.

Children^a^ constitute an important population sub-group in health research, and their growing inclusion in research has led to questions about how paediatric consent operates in practice. This is partly attributable to an expanding body of evidence that indicates tremendous gaps, variability, and apparent inconsistency in the content of consent forms for health research, even for similar studies or at different sites within the same study [1-8]. This may be due to, in part, new challenges raised by health research that are not addressed or settled in current guidelines. Another reason, often stated by research ethics boards (REBs) themselves, may be sensitivity to local concerns of participant communities, administrations, and cultures [9]. While some flexibility and diversity is warranted, consent form variability and inconsistency should be scrutinised for several reasons. First, large-scale study forms that do not consider the growing importance that many significant research funders place on broad data sharing may impede data flow through varying modalities of consent, data or sample coding, and data or sample transfer policies [10]. Second, from an ethical viewpoint, inconsistency and a lack of harmonisation may unevenly protect research participants [11]. Third and most crucially, it can undermine the trust that parents ^b^, children, researchers and society place in research enterprises, and ultimately cause harm to children’s rights [12,13].

Considering the rapid research developments in areas such as biobanking, longitudinal studies, gene-gene or gene-environment studies, and exome- or genome-wide association studies, it is important to examine the approaches in consent forms that address several key issues in paediatric research that the literature identifies as emerging [14-24], namely those arising under the domain of: (1) the scope of consent of the parent and/or child; (2) risks and benefits; (3) the right of withdrawal; (4) return of research results and incidental findings; (5) privacy and confidentiality; and (6) retention of and access to the child’s data and/or samples.

These issues have significant impact on the content of the informed consent forms and its process. In response to the growing research in the field of paediatrics, we hope to draw attention to emerging ethical issues in paediatric research where further harmonisation could surface. Our objective in the first part of the analysis is a) to find out how much information refers to these emerging issues, and b) to evaluate the quality using a structured checklist based on the best practices. Since we noted a lack of consistency, in the second part, we propose some best practices for the development of the consent forms. This, we contend, is a pressing need because children are vulnerable and require protection [25], and because their specific health interests via research require promotion. Addressing the emerging issues in paediatric research consent forms will facilitate harmonised and yet contextualised approaches, hopefully promoting a safer and healthier world for children.

## Methods

### Sample identification

REB-approved assent and consent forms dated 2008–2011 for paediatric research from across Canada were collected. Members of Canada’s Maternal Infant Child and Youth Research Network (MICYRN), which consists of 17 child health research organisations at academic health centres affiliated with universities or medical schools in Canada, were contacted and asked to provide copies of their REB-approved informed consent forms. In addition, we searched websites of large organisations engaged in paediatric research across Canada using our personal knowledge of ongoing research projects at leading hospitals and research institutes. By way of purposive sampling, we deliberately collected consent forms arising from observational, genetic, longitudinal, and clinical trial studies. Through the end of 2011, we collected and reviewed 65 forms.

**Figure 1:**
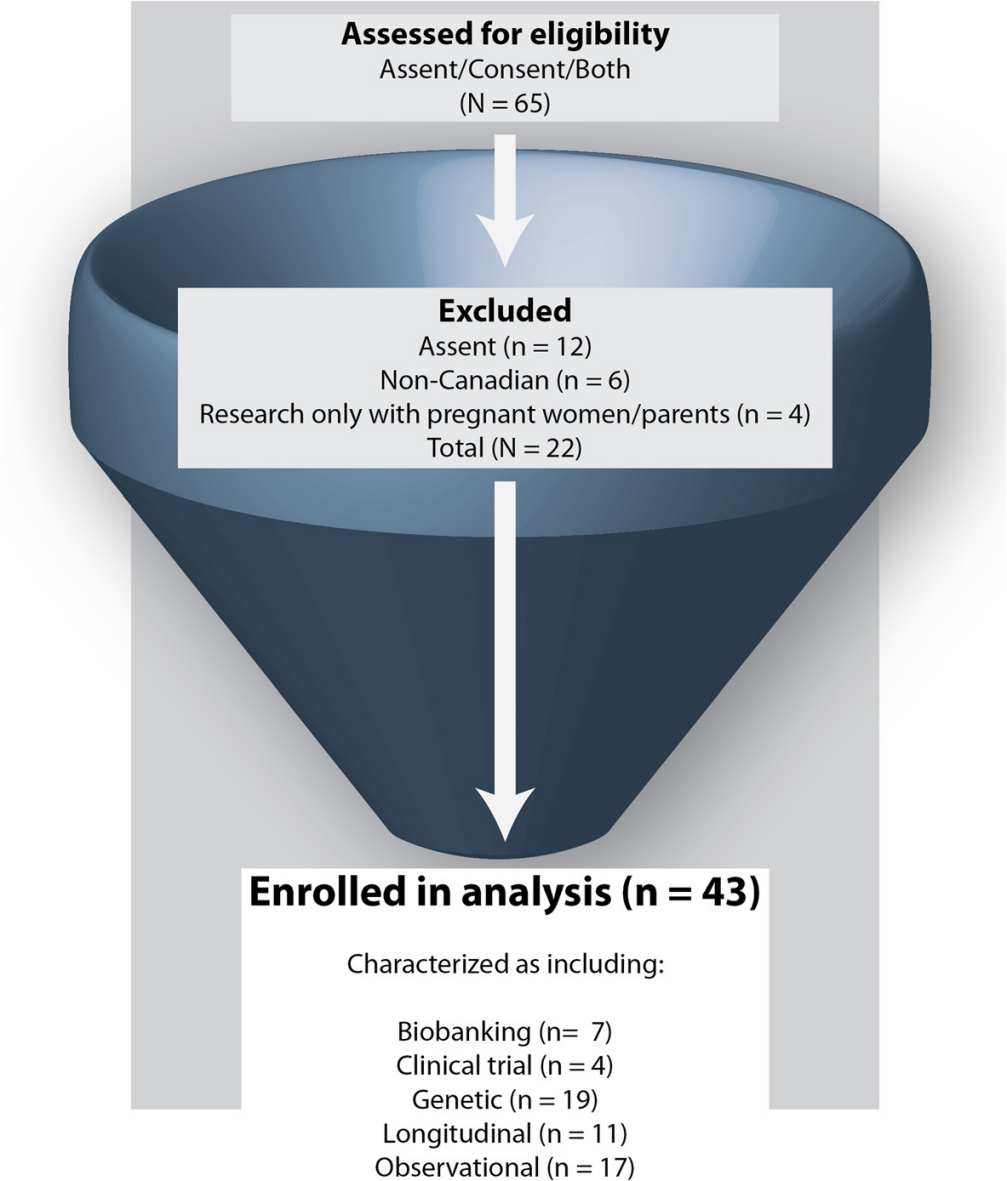
Flow chart with the results of the search strategy.

Figure 1 depicts the inclusion and exclusion criteria. We excluded assent forms (n = 12) since they are addressed only to children, are somewhat distinct from consent forms, and will be the subject of a separate paper. We also excluded non-Canadian forms (n = 6) and forms (n = 4) that consisted only of research conducted with pregnant women and/or parents and not with neonates or children. The resulting data set consisted of 43 documents that met our inclusion criteria and addressed at least one of our emerging issues. They were selected from major academic research centres or public health organisations that included forms (with some overlap) for studies that included biobanks, clinical trials, longitudinal studies, observational studies, as well as paediatric research consent form templates (n = 16) drafted by research institutions, hospitals, or government agencies.

### Data abstraction

**Figure 2:**
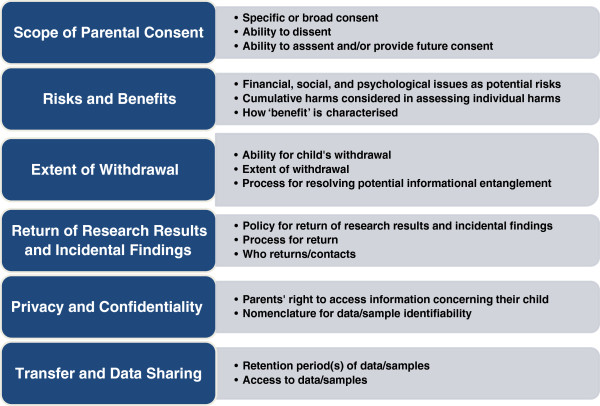
Summary of emerging issues in six domains.

Following a modified qualitative thematic content analysis [26], the content of the consent forms was coded using an ‘a priori’ coding approach based on emerging issues falling under six domains (Figure 2). These domains were framed as determining whether: (1) the scope of parental consent allows for a child’s assent, dissent, or future consent; (2) the concepts of risk and benefit incorporate the child’s psychological and social perspective; (3) a child’s ability to withdraw is respected and to what extent withdrawal is permitted; (4) the return of research results includes individual results and/or incidental findings and the processes involved therein; (5) privacy and confidentiality concerns adequately address the child’s perspective and whether standard data and/or sample identifiability nomenclature is used; and (6) retention of and access to paediatric biological samples and associated medical data are addressed. These domains (and the sub-issues within each) were chosen to provide a benchmark by which to judge how the issues were addressed in the consent forms and because they represent, in our opinion and experience as paediatric health ethics researchers, the most debated and unresolved in the field. Two reviewers independently screened the consent forms (ESD and ML). Any discrepancies were reconciled and checked by another evaluator (DA). The analysis consisted of a review of the written information in the forms, which was extracted and presented in tables.

## Results

The results of our research are summarised in Table 1 (see also Additional file 1).

**Table 1:** Emerging issues in paediatric research consent forms

**Emerging issues in paediatric research discussed in consent forms**	**N total (43)**	**%**
**1. Consent**		
Scope of parental consent		
Specific	13	30
Broad	18	42
Broad or Specific (option)	2	5
Not addressed	10	23
Child’s ability to dissent		
Addressed	19	44
Not addressed	24	56
Possibility for child’s assent and/or future consent		
Assent or future consent addressed	22	51
Neither addressed	21	49
**2. Risks and benefits**		
Financial, social or psychological issues as potential risks	11	26
Cumulative harms considered	3	7
How ‘benefit’ characterised		
Indirect	29	67
Unspecified	11	26
Direct or combination of direct/indirect	3	7
**3. Extent of withdrawal**		
Ability for child to withdraw		
Addressed	28	65
Not addressed	15	35
Extent of withdrawal		
All data/samples destroyed	7	16
No further use of data/samples	1	2
Retention of data/samples collected to date	3	7
Not addressed or unspecified	32	74
Process for handling parental-child disagreement on withdrawal	0	0
**4. Return of research results and incidental findings**		
Not addressed	17	40
Addressed	26	60
No return	6	14
General/aggregate results return	7	16
Individual results return	7	16
General/aggregate and individual results return	6	14
Any return of results that include incidental findings (with or without option)	8	19
**5. Privacy and confidentiality**		
Scope of parental right to access information concerning their child	0	0
Nomenclature for data/sample identifiability		
Unspecified	14	33
Coded	25	58
Anonymised	4	9
**6. Transfer and data sharing**		
Retention period(s) of data/samples		
Indefinite	12	28
Specified time periods	18	42
Combination of indefinite and specified time periods, depending on whether material is data or samples	4	9
Not addressed	9	21
Access to data/samples		
No transfer	2	5
Disclosure that data/samples may be transferred to another location	17	40
Disclosure and discussion of procedure for external data/sample transfer	4	9
Not addressed	20	47

### Consent

Thirty percent of the forms used specific consent (i.e. the participant is informed in a detailed fashion of the research objectives, procedures, risks, benefits, and future uses of data and samples collected), while 42% used broad consent (i.e. the participant is informed that data and samples may be used in future, unspecified studies). The remainder either did not specify the scope, as they were template forms, or presented an option for specific or broad consent. Fifty-six percent of the forms did not address a child’s ability to dissent; 49% of the forms addressed neither assent nor the potential for future re-consent at the legal age of majority, while 30% addressed assent with qualifications such as a specific age.

### Risks and benefits

Only a quarter of the forms (25.6%) addressed financial, social, or psychological issues in the context of potential risks. One form – a template – addressed all three issues. Three forms explicitly considered cumulative harms for the child participant. To classify ‘benefit’, we distinguished a direct benefit (i.e. a tangible positive outcome whereby there is an intervention intended to prevent, diagnose, or treat illness or injury) and indirect benefit (i.e. benefits to other children of the same age or with the same condition, or benefits that are not related to the research objectives as such that could include gifts or payments). Sixty-seven percent of the forms expressed indirect benefits to research participants, often in the form of altruistic notions of helping society benefit from greater knowledge of a particular disease or childhood development. One form related to a clinical genetic study indicated a direct benefit to the child. Two forms (in the context of clinical trials) indicated both a potential direct benefit to the child as well as indirect benefits to society or other paediatric patients in the future.

### Withdrawal

Sixty-five percent of the forms addressed a child’s possible ability to withdraw, none of which imposed qualifications such as demonstrable competence and maturity in making such a decision. Other forms, however, addressed the right to withdraw only to the parents and not to the child. For example, one consent form for a study to evaluate the safety and immunogenicity of a vaccine in children aged 6 months to 18 years stated that: ‘Taking part in this study is entirely your choice. You may decide not to enrol your child and you may withdraw your child from the study at any time.’ Indeed, no mention was made of the right of the child, even in adolescence or teenage years, to independently withdraw, and there was no assent form associated with this consent form in which such information could have been included. Regarding the extent of withdrawal, only 16% stated that all unused samples and/or data would be destroyed upon a request to withdraw; two forms stated that there would be no further use of the data and/or samples, and one stated that data collected up until the declaration of withdrawal would not be removed. None of the forms disclosed a process for handling ‘informational entanglement’, i.e. parental disagreement on withdrawal between themselves or with the mature minor.

### Return of research results and incidental findings

Sixty percent of the consent forms addressed the possible return of research results. However, there was no consensus, with approximately 15% of the total number of forms each indicating: no return, return of general/aggregate results, or, of individual research results (including incidental findings), or, the return of both general/aggregate and individual research results. There were also various processes involved for the return, with some studies opting for a liaison with an independent laboratory to confirm an incidental finding, as well as providing the option of genetic counsellors to explain the implications of the finding. All of the forms addressing return of research results (other than a no return policy) revealed that the researchers would return the results, though one clinical trial form stated that the patient’s doctor would possibly return individual results and incidental findings. Most of the genetic study or biobank consent forms that mentioned incidental findings stated that in addition to disclosure of research results by the researchers, participants had the option for an independent genetic clinic to confirm test results and for genetic counsellors to discuss such findings or genetic test results.

### Privacy and confidentiality

None of the forms disclosed any information about a parent’s qualified or unqualified right to access information concerning their child, though indirectly related, one form discussed the situation of a child’s pregnancy while enrolled. One-third of the forms did not specify a particular manner of protecting data and sample identifiability, other than a general statement of a commitment to keep all information confidential and protected, but 58% used the word ‘coded’ for data and/or samples. Only 9% stated that data and/or samples would be anonymised.

### Retention of and access to child’s data and/or samples

Forty-two percent of the forms disclosed that data/samples would be retained and disclosed the period of time (including if it was an indefinite period), or in the case of template forms, stated that researchers should specify the retention period(s). Twenty-eight percent stated that data/samples would be retained, but did not specify a period, while 9% disclosed that samples would be held for a period of time (in these cases, indefinitely), but did not specify a period for data retention. Lastly, 47% did not discuss whether data/samples would be transferred to another location (e.g. province, country) during or after the study. Forty percent disclosed that data/samples could be transferred to another location, and 9% went beyond simple disclosure and also stated the transfer process of the data/samples. Two forms explicitly stated that there would be no transfer of data/samples outside of the study site.

## Discussion

The results indicate incongruent approaches to addressing emerging issues, if indeed they are addressed at all. There are several explanations for this, and each issue may manifest different rationales for variability. With respect to consent, for example, it is generally accepted that the failure to address in some capacity a child’s assent and then later consent as they mature (if the research project continues over a prolonged period) can undermine the integrity of the project and create schisms in the current and future protection of a child’s wellbeing and developing autonomy. These issues are of particular importance in longitudinal and biobanking studies that intend to use samples and data indefinitely. But re-contact, perhaps in studies not specifically longitudinal in nature, may be regarded as unforeseen, unfeasible, or unrealistic. Hence, an explanation for the common implementation of ‘broad consent’ in the consent forms we reviewed may be that it is viewed as the most practical, efficient, and appropriate scope of consent, provided the samples and data are coded rather than anonymised so that re-contact is possible. It should be noted that broad consent was largely seen, as expected, in the longitudinal and biobanking research studies.

That the majority of the consent forms we analyzed did not address cumulative or non-physical risks may speak to the definitional ambiguity of ‘risk’ and the tendency to focus on physical risks. Risk is defined in Canada’s 2010 *Tri-Council Policy Statement* (TCPS) [27]^c^ as ‘a function of the magnitude or seriousness of the harm, and the probability that it will occur’ (TCPS, Ch. 2B), but this does not address the ambit of harm. In paediatric research, harm may encompass psychological, social, financial, and community risks, particularly in genetic, biobanking, and longitudinal research. This is more than an ethical concern. In Canada, full disclosure of risks in research is legally required [28,29]. Along the same lines, given that most forms stated that indirect benefits (e.g. a societal benefit from biomedical advancement) would be achieved, one must conclude that the research projects entailed no more than ‘minimal risk’ in order to receive REB approval, as required by the TCPS (Art. 4.6). Yet, it remains an open question whether these projects truly pose a minimal risk if they do not disclose non-physical risks, consider cumulative risks, or consider risks from the perspective of the child whose perspective may differ drastically depending on age [30].

The lack of specificity in some of the forms that the child could withdraw rather than the parent providing the authorisation, and the lack of a resolution procedure for withdrawal conflicts between children and parents, may be due to normative guidelines that generally *encourage*, rather than require, researchers to respect a child’s decision to withdraw from research if the child has the capacity and maturity to make an independent choice [19,31]. The consent forms that failed to clearly state to the parents that withdrawal may not in fact be absolute could be due to instances where data and samples are irretrievably de-linked to an identifiable person (i.e. they are anonymised). Yet, almost no forms indicated anonymisation was involved, so this is only a partial explanation. This also leaves open the question as to whether parents or children are aware that should they later change their mind about participating in a study that has anonymised their data or samples, destruction of their data or samples is no longer possible as they cannot be identified.

The return of research results has garnered significant discussion, especially with the advent of whole genome and exome sequencing [20,32,33]. Incidental findings, defined as ‘unanticipated discoveries made in the course of research but that are outside the scope of the research’ (TCPS Article 3.4), are becoming increasingly important as data-intensive science expands and next generation sequencing technologies are employed [34,35]. Currently, there is neither national nor international consensus on the treatment of incidental findings in paediatrics, and the consent forms we reviewed reflect this variability and continuing lack of consensus, with respect to both return of research results and incidental findings. In cases where results reveal a clinically significant condition for which there is current treatment or prevention, the parents cannot refuse to know and the child’s right to medical care prevails [14,21,36]. In other situations, researchers – and parents – may opt to wait until the child is mature enough or reaches majority before disclosing research results if the results are not materially relevant until the child reaches adulthood. Either way, the potential for these situations, especially if their possibility is known at the time of consent, should be explained in the consent form and further elaborated upon by the individual obtaining consent. Similarly, consent forms should be clear if the child’s consent or assent to receiving the information would be sought so as to not compromise the child’s ‘right to an open future’ [37], and if the ‘right not to know’ necessarily includes the right not to have information included in the medical record if it entails an actionable result.

It is a positive sign that most of the forms we analysed provided standardised nomenclature for sample or data identifiability (e.g. ‘coded’), since terminological confusion has long been an issue [38]. However, standardised nomenclature may be seen as only the first step to addressing privacy and confidentiality concerns. Challenges to privacy and confidentiality are amplified by genomics and other biomedical research projects [39], which are often internationally collaborative and engage in perpetual data linkage across jurisdictions. Biobanking or genetic research consent forms may need to declare that neither anonymised nor coded data and samples can guarantee privacy, as knowledge of even a small number of genetic variants can lead to matching of samples to individuals with a high level of confidence (we did not observe consent forms that disclosed this potential privacy risk).

All of the consent forms assumed parents could access research-related information about their child. And yet, multiple tensions can arise between the child’s privacy interests and the parents’ general legal right to their child’s health information [40-42]. For example, a child may not want her parents to know about a pregnancy test result or habitual drug use, but this desire could conflict with legal duties of parental access to health information that compel a researcher to disclose such information. At a minimum, consent forms should disclose the kind or extent of information communicated to the parent and information which shall require the child’s consent.

Lastly, while ethical norms generally support transfer of data and samples with certain safeguards, there is continuing debate about the parameters of that transfer and the various organisational safeguards, technological measures, and physical measures that should be adopted and updated – and disclosed – in the consent form. Research participants remain woefully uninformed of the transfer of their samples and data, particularly when it may carry a commercial use [43]. Devising and disclosing a method for listing all approved projects that are accessing the data and/or samples could alleviate this. While it may be the case that all of the consent forms we analysed that did not address transfer of data and samples simply did not envision transfer, in the absence of explicit disclosure that ‘no transfer will occur’, uncertainty remains and creates the risk of future ethical concerns with maturing children.

## Study limitations

Our study had some limitations. It relied on our purposive sampling derived from Canada, which may not be generalisable to other countries. Our data focuses on emerging ethical issues rather than on the context of the consent process or on the quality of consent form information and its actual comprehension by participants. These are two critically important topics discussed elsewhere in the literature [44-46]. Similarly, the amount and quality of information extracted from the consent forms regarding these six issues cannot provide an estimated average that is generalisable to all consent forms, though the range of issues in our purposive sampling reveals important gaps. Some types of consent forms may have been underrepresented, such as qualitative research or community setting research. There are other emerging issues impacting the consent process that were not considered in this study due to resource constraints, but are worthy of future reviews, such as the issue of incentives for participating in research. This is an area that is rapidly becoming a point of controversy; offering monetary payments, gift certificates, or toys to parents or children who participate in research touch on ethical issues of undue influence and voluntariness [47-50]. Despite these limitations, this research addresses important gaps in the literature by incorporating evidence regarding emerging ethical issues that will in turn improve the usefulness of paediatric consent forms.

## Towards best practices

As our Discussion section notes, divergence in these six distinct domains in paediatric research reflects various factors. The rapidly evolving nature of science and technology can hamper the ability of researchers and REBs to keep abreast of socio-ethical discourse surrounding the inclusion of various types of emerging issue information. But, it is insufficient to only identify problem areas and explanations in the current environment. Remedies should be offered as well. Therefore, we suggest some best practices that can improve consent forms and facilitate harmonised and yet contextualised approaches to ethical norms in paediatric research.

We opt for best practices deliberately. In the modern, diverse research environment, where there is a plethora of possibilities (sometimes overlapping), not all paediatric research is alike. Designing a standard template for consent forms other than for the most basic provisions would restrict the flexibility needed to accommodate scientific developments and local contexts [39]. Best practices, however, can serve as useful general guidance to researchers when they design their paediatric research projects and draft consent forms. They can also help make REBs more aware of key issues and better scrutinise consent forms for ethical compliance, and can help ensure REBs maintain a flexible and innovative approach to template consent forms. A set of best practices can encourage dialogue between REBs and researchers to ensure that each informs the other of emerging issues and to not rigidly adhere to document templates for wording and formatting. Finally, best practices can help catalyse the growing importance of paediatric research and serve as a blueprint for further development of standards and guidance, such as online educational and practical tools to enhance understanding of the emerging issues.

Building on the *Best Practices for Health Research Involving Children and Adolescents*[14], we propose a non-exhaustive list of what we consider to be best practices for drafting consent forms that address the emerging issues discussed in this article (Table 2).

**Table 2:** Best practices for drafting paediatric research consent forms in Canada

**Emerging issue**	**Best practices**
**Scope of parental consent**	
Broad consent	· The possibility of future, unspecified research uses should be mentioned prior to obtaining consent and the consent form should be worded accordingly.
Ability to assent/provide future consent	· When the child is considered to be legally able to provide consent, consent should be renewed, if feasible.
	· Where feasible, data and/or samples should be coded (not anonymised) in order to allow researchers to maintain contact with the child.
Ability to dissent	· The possibility of a child’s right to dissent, provided there is an ability to understand the significance of research or his/her role in it, should be disclosed.
**Risks and benefits**	
Financial, social, and psychological issues	· Consideration of potential harms must include physical as well as psychological, social or financial harms.
Cumulative harms considered in assessing individual harms	· Cumulative harms should be considered.
How ‘benefit’ is characterised	· Risks and benefits should be considered from the child’s perspective.
**Withdrawal**	
Ability for withdrawal	· The child’s ability to withdraw should be explicitly disclosed, as well as any circumstances that might limit the ability (e.g. if immediate withdrawal could harm the child).
Extent of withdrawal	· The *extent* of the ability to withdraw should be explicitly disclosed (e.g. if data and/or samples are anonymised, the consent form should state that withdrawal is not feasible).
Informational entanglement	· The potential for a child and parents to disagree about whether to withdraw, and its potential impact on the research project, should be described.
**Return of research results and incidental findings**	
The potential and process for returning research results and incidental findings	· The potential for disclosing research results and incidental findings, as well as its process (including who discloses and the possibility for entitlement to non-disclosure), should be described.
Returning actionable individual results and incidental findings	· Individual research results and incidental findings that have clinical significance should be communicated to the child and/or parents when either prevention or treatment is available during childhood, and with adequate counselling provided. The interconnected nature of the potential risks and benefits of such communication should be disclosed.
Duty to receive information	· Parents should be made aware that they will receive clinically significant information about conditions that are preventable or treatable during childhood.
**Privacy and confidentiality**	
Parents’ right to access information concerning their child	· In research projects that collect and use particularly sensitive information, such as pregnancy status, drug use, or sexual history, consent forms should disclose what information will and will not be communicated to parents, and which information disclosure requires the child’s consent.
Nomenclature for data/sample identifiability	· Standardised sample identifiability terminology should be used: coded (including single-coded and double-coded), anonymised, and anonymous.
	· Biobanking or genetic research consent forms should declare that anonymised or coded data and samples cannot absolutely guarantee privacy.
**Retention of and access to data/samples**	
Retention period(s) of data/samples	· Consent forms should clearly distinguish between what is a legally required data/sample retention period and a retention period decided upon by the researcher.
Access to data/samples	· The policies and procedures for access to data and/or samples should be disclosed.
	· These policies and procedures should consider the privacy impact (both to the parents and child) of access to coded or anonymised information, including: organisational safeguards, technological measures, physical measures, and ethics oversight.
	· If feasible, researchers should disclose a method for listing all approved projects that are accessing the data and/or samples.

## Conclusions

This article assesses Canadian paediatric consent forms in light of emerging ethical issues in paediatric consent practices and identifies many gaps and inconsistencies among the forms. Attention to the best practices could make a big difference. To this end, we acknowledge that informed consent is infinitely more complex than ethics guidelines or law imply. The more those in the research community recognise that information disclosed to a participant depends on context (e.g. study project, location, resources) and must go beyond mere duties of disclosure to actually achieve *understanding*[51], the further researchers and REBs can move towards ensuring there is genuine research participation, and indeed, engagement [52-54]. To help get there, future research should couple a thematic analysis of paediatric consent or assent forms with surveys of views and experiences of parents and children so as to offer a more holistic approach to evaluating the strengths and weaknesses of these forms.

At the same time, consent forms cannot and should not include all issues under the sun, lest information overload ensue. More information is not always better information; indeed, consent form length may not materially affect the quality of informed consent or consent rate [55,56]. While some may perceive these best practices as cumbersome additions to already unwieldy consent forms, appreciation for contextualisation and brevity must be distinguished from inappropriate omissions or unreasonable and unpredictable ethical standards. We hope that the best practices listed in Table 2 will be a useful guide for both the drafters of consent forms and for REBs.

Ultimately, organisations, funding agencies, as well as researchers and REBs, must work together to develop a well-forged, dynamic ethical and legal toolbox to ensure that consent forms disclose a sufficiently uniform level of understandable information, including potentially contentious issues, so that parents and children (to the extent they are capable) can make an informed decision together [57]. Attention to best practices will improve research collaboration, provide workable tools for researchers and ethics boards, and improve the ethical tensions that can occur in the tri-partite relationship between the child, parents, and researchers. This enables us to ensure that the most important participants in this process – children – are fully protected, respected and given the opportunity to grow up in a healthier and safer world.

## Endnotes

^a^ For this article, we adopt the definition of ‘child’ in Article 1 of the UN *Convention on the Rights of the Child* (1989): ‘…a child means every human being below the age of eighteen years unless under the law applicable to the child, majority is attained earlier.’

^b^ For this article, ‘parent(s)’ also includes legal representative(s) and legal guardian(s).

^c^ Paediatric researchers and their institutions who receive funds from the three major Canadian federal funding agencies (Canadian Institutes of Health Research, Sciences and Engineering Research Council, and Social Sciences and Humanities Research Council) must ensure that their consent forms conform to ethical standards established by the TCPS. Certain health and social service institutions in provinces also have policies endorsing the TCPS (e.g. the Ministère de la Santé et des Services sociaux du Québec).

## Abbreviations

MICYRN: Maternal Infant Child and Youth Research Network; REB: Research ethics board; TCPS: Tri-Council Policy Statement.

## Competing interests

The authors have no competing interests.

## Authors’ contributions

All authors conceived the study and participated in its design. LB, DA, and BMK collected the raw data. ESD, LB, and DA coded the data. BMK helped in the coordination of the study. ESD, LB, and DA performed data analysis. ESD drafted the manuscript. All authors were involved in the editing. All authors have read and approved the final manuscript.

## Ethical approval

No ethical approval was sought in connection with the study as, in accordance with Canadian ethics guidelines, there was no research involving human subjects or research on information containing personally identifiable information.

## Pre-publication history

The pre-publication history for this paper can be accessed here:

http://www.biomedcentral.com/1472-6939/14/5/prepub

## Supplementary Material

Additional file 1 Table of results for consent forms in addressing emerging issues. Click here for file
