# Supplementary material for: Emerging issues in paediatric health research consent forms in Canada: working towards best practices
Source: BMC Med Ethics. 2013 Jan 30;14:5. doi: 10.1186/1472-6939-14-5 (PMC3571865; doi:10.1186/1472-6939-14-5)
Supplement: Additional file 1 — Table of results for consent forms in addressing emerging issues. [file 1472-6939-14-5-S1.doc]

List of consent forms analysed.

| **Case Number** | **Year of form** | **Type of study** |
| --- | --- | --- |
| 1 | 2007 | Template |
| 2 | 2007 | Template |
| 3 | 2008 | Genetic |
| 4 | 2008 | Longitudinal, Observational |
| 5 | 2008 | Longitudinal, Genetic, Environmental testing, Observational |
| 6 | 2008 | Longitudinal, Genetic, Environmental testing, Observational |
| 7 | 2008 | Longitudinal, Genetic, Environmental testing, Observational |
| 8 | 2008 | Longitudinal, Genetic, Environmental testing, Observational |
| 9 | 2008 | Genetic, Biobanking |
| 10 | 2008 | Disease aetiology, Environmental testing, Longitudinal, Observational |
| 11 | 2008 | Template |
| 12 | 2008 | Template |
| 13 | 2009 | Template |
| 14 | 2009 | Template |
| 15 | 2009 | Biobanking, Genetic, Observational |
| 16 | 2009 | Disease aetiology, Genetic |
| 17 | 2009 | Environmental testing, Longitudinal, Observational |
| 18 | 2009 | Biobanking, Disease aetiology, Genetic |
| 19 | 2009 | Biobanking, Disease aetiology, Genetic |
| 20 | 2009 | Genetic |
| 21 | 2009 | Genetic, Environmental testing, Longitudinal, Observational |
| 22 | 2009 | Genetic, Environmental testing, Longitudinal, Observational |
| 23 | 2009 | Genetic, Environmental testing, Longitudinal, Observational |
| 24 | 2009 | Genetic, Environmental testing, Longitudinal, Observational |
| 25 | 2009 | Template |
| 26 | 2010 | Genetic |
| 27 | 2010 | Genetic |
| 28 | 2010 | Template |
| 29 | 2010 | Biobanking, Genetic |
| 30 | 2010 | Biobanking |
| 31 | 2010 | Template |
| 32 | 2011 | Genetic |
| 33 | N/A | Template |
| 34 | N/A | Clinical trial, Observational |
| 35 | N/A | Template |
| 36 | N/A | Template |
| 37 | N/A | Template |
| 38 | N/A | Template |
| 39 | N/A | Template |
| 40 | N/A | Clinical trial, Observational |
| 41 | N/A | Template |
| 42 | 2010 | Clinical trial |
| 43 | 2011 | Clinical trial |

Consent issues.

| **Case Number** | **Scope of Parental Consent** | **Child’s Ability to Dissent** | **Possibility for Child’s Assent and/or Future Consent** |
| --- | --- | --- | --- |
| 1 | Unspecified | Not addressed | Assent addressed  (with qualifications) |
| 2 | Unspecified | Addressed | Assent addressed  (with qualifications) |
| 3 | Specific | Not addressed | Assent and future consent  not addressed |
| 4 | Specific | Not addressed | Assent and future consent  not addressed |
| 5 | Broad | Addressed | Assent and future consent  not addressed |
| 6 | Broad | Addressed | Assent and future consent  not addressed |
| 7 | Broad | Addressed | Assent and future consent  not addressed |
| 8 | Broad | Addressed | Assent and future consent  not addressed |
| 9 | Broad | Not addressed | Assent addressed  (with qualifications) |
| 10 | Specific | Not addressed | Assent and future consent  not addressed |
| 11 | Unspecified | Not addressed | Assent addressed  (with qualifications) |
| 12 | Broad | Not addressed | Assent addressed  (with qualifications) |
| 13 | Unspecified | Addressed  (with qualifications) | Assent addressed (without qualifications) |
| 14 | Unspecified | Not addressed | Assent and future consent  not addressed |
| 15 | Broad | Not addressed | Assent and future consent  not addressed |
| 16 | Specific | Addressed  (with qualifications) | Assent addressed (without qualifications) |
| 17 | Specific or Broad (option) | Not addressed | Assent and future consent  not addressed |
| 18 | Specific | Addressed | Assent addressed (with qualifications) |
| 19 | Broad | Not addressed | Assent addressed (without qualifications) |
| 20 | Specific | Addressed | Assent addressed (with qualifications) |
| 21 | Broad | Not addressed | Assent and future consent  not addressed |
| 22 | Broad | Not addressed | Assent and future consent  not addressed |
| 23 | Broad | Not addressed | Assent and future consent  not addressed |
| 24 | Broad | Not addressed | Assent and future consent  not addressed |
| 25 | Specific or Broad (option) | Addressed | Assent addressed (without qualifications) |
| 26 | Broad | Not addressed | Assent and future consent  not addressed |
| 27 | Broad | Not addressed | Assent and future consent  not addressed |
| 28 | Specific | Addressed | Assent addressed (without qualifications) |
| 29 | Specific | Not addressed | Assent addressed (with qualifications) |
| 30 | Broad | Not addressed | Assent addressed (without qualifications) |
| 31 | Unspecified | Addressed  (with qualifications) | Assent and future consent addressed |
| 32 | Broad | Not addressed | Assent and future consent addressed |
| 33 | Unspecified | Addressed | Assent addressed (with qualifications) |
| 34 | Specific | Not addressed | Assent and future consent  not addressed |
| 35 | Specific | Addressed | Assent addressed (with qualifications) |
| 36 | Specific | Addressed | Assent and future consent  not addressed |
| 37 | Specific | Addressed | Assent and future consent  not addressed |
| 38 | Unspecified | Addressed | Assent addressed (with qualifications) |
| 39 | Unspecified | Not addressed | Assent addressed (with qualifications) |
| 40 | Specific | Not addressed | Assent and future consent  not addressed |
| 41 | Unspecified | Addressed | Future consent addressed |
| 42 | Broad | Addressed | Assent addressed (with qualifications) |
| 43 | Broad | Not addressed | Assent and future consent  not addressed |

**Scope of Parental Consent**

Specific: 13/43
Broad: 18/43
Broad or Specific (option): 2/43
Unspecified: 10/43

**Child’s Ability to Dissent**

Not addressed: 24/43
Addressed (with qualifications): 3/43
Addressed (without qualifications): 16/43

**Possibility for Child’s Assent and/or Future Consent**

Assent and future consent not addressed: 21/43
Assent addressed (with qualifications): 13/43
Assent addressed (without qualifications): 6/43
Assent and future consent addressed: 2/43
Future consent addressed: 1/43

Risk and benefit issues.

| **Case Number** | **Financial, Social and Psychological Issues as Potential Risks** | **Cumulative Harms Considered in Assessing Individual Harms** | **How ‘Benefit’ is Characterised** |
| --- | --- | --- | --- |
| 1 | Psychological risks addressed | Not addressed | Unspecified |
| 2 | Not addressed | Cumulative harms considered | Unspecified |
| 3 | Not addressed | Not addressed | Direct |
| 4 | Not addressed | N/A | Indirect |
| 5 | Not addressed | Not addressed | Indirect |
| 6 | Not addressed | Not addressed | Indirect |
| 7 | Not addressed | Not addressed | Indirect |
| 8 | Not addressed | Not addressed | Indirect |
| 9 | Not addressed | N/A | Indirect |
| 10 | Not addressed | Not addressed | Indirect |
| 11 | Social and psychological risks addressed | Not addressed | Unspecified |
| 12 | Not addressed | Not addressed | Unspecified |
| 13 | Psychological risks addressed | Not addressed | Unspecified |
| 14 | Not addressed | Not addressed | Unspecified |
| 15 | Not addressed | N/A | Indirect |
| 16 | Not addressed | N/A | Indirect |
| 17 | Psychological risks addressed | Not addressed | Indirect |
| 18 | Social and psychological risks addressed | N/A | Indirect |
| 19 | Not addressed | N/A | Indirect |
| 20 | Social and psychological risks addressed | N/A | Indirect |
| 21 | Not addressed | Not addressed | Indirect |
| 22 | Not addressed | Not addressed | Indirect |
| 23 | Not addressed | Not addressed | Indirect |
| 24 | Social and psychological risks addressed | Not addressed | Indirect |
| 25 | Not addressed | N/A | Unspecified |
| 26 | Psychological risks addressed | Not addressed | Indirect |
| 27 | Psychological risks addressed | N/A | Indirect |
| 28 | Not addressed | Not addressed | Indirect |
| 29 | Not addressed | N/A | Indirect |
| 30 | Not addressed | N/A | Indirect |
| 31 | Financial, social and psychological risks addressed | Cumulative harms considered | Unspecified |
| 32 | Not addressed | Cumulative harms considered | Indirect |
| 33 | Not addressed | Not addressed | Unspecified |
| 34 | Not addressed | Not addressed | Indirect |
| 35 | Not addressed | N/A | Indirect |
| 36 | Not addressed | N/A | Indirect |
| 37 | Not addressed | N/A | Indirect |
| 38 | Not addressed | Not addressed | Unspecified |
| 39 | Not addressed | Not addressed | Unspecified |
| 40 | Not addressed | Not addressed | Direct & Indirect |
| 41 | Not addressed | Not addressed | Indirect |
| 42 | Social risks addressed | Not addressed | Direct & Indirect |
| 43 | Not addressed | Not addressed | Indirect |

**Financial, Social and Psychological Issues as Potential Risks**

Financial, social and psychological risks addressed: 1/43
Social and psychological risks addressed: 4/43
Social risks addressed: 1/43
Psychological risks addressed: 5/43
Not addressed: 32/43

**Cumulative Harms Considered**

Cumulative harms considered: 3/43

**How ‘Benefit’ is Characterised**

Direct benefit: 1/43
Indirect benefit: 29/43
Direct & indirect benefit: 2/43
Unspecified: 11/43

Withdrawal issues.

| **Case Number** | **Existence of an Ability for the Child to Withdrawal** | **Extent of Child’s Ability to Withdraw** | **Process for Handling Parental-Child Disagreement on Withdrawal** |
| --- | --- | --- | --- |
| 1 | Not addressed | Not addressed | Not addressed |
| 2 | Addressed (without qualifications) | Unspecified | Not addressed |
| 3 | Addressed (without qualifications) | All unused samples destroyed | Not addressed |
| 4 | Not addressed | Not addressed | Not addressed |
| 5 | Addressed (without qualifications) | Not addressed | Not addressed |
| 6 | Addressed (without qualifications) | Not addressed | Not addressed |
| 7 | Addressed (without qualifications) | Not addressed | Not addressed |
| 8 | Addressed (without qualifications) | Not addressed | Not addressed |
| 9 | Addressed (without qualifications) | No further use of data and samples | Not addressed |
| 10 | Not addressed | Not addressed | Not addressed |
| 11 | Addressed (without qualifications) | Unspecified | Not addressed |
| 12 | Addressed (without qualifications) | Unspecified | Not addressed |
| 13 | Addressed (without qualifications) | Unspecified | Not addressed |
| 14 | Addressed (without qualifications) | Unspecified | Not addressed |
| 15 | Not addressed | Not addressed | Not addressed |
| 16 | Not addressed | Not addressed | Not addressed |
| 17 | Not addressed | Not addressed | Not addressed |
| 18 | Addressed (without qualifications) | No further use of data | Not addressed |
| 19 | Addressed (without qualifications) | All data and unused samples destroyed | Not addressed |
| 20 | Addressed (without qualifications) | All unused samples destroyed  Data retained | Not addressed |
| 21 | Not addressed | Not addressed | Not addressed |
| 22 | Not addressed | Not addressed | Not addressed |
| 23 | Not addressed | Not addressed | Not addressed |
| 24 | Not addressed | Not addressed | Not addressed |
| 25 | Not addressed | Not addressed | Not addressed |
| 26 | Addressed (without qualifications) | All data and unused samples destroyed | Not addressed |
| 27 | Addressed (without qualifications) | All data and unused samples destroyed | Not addressed |
| 28 | Not addressed | Not addressed | Not addressed |
| 29 | Addressed (without qualifications) | Not addressed | Not addressed |
| 30 | Addressed (without qualifications) | All data and unused samples destroyed | Not addressed |
| 31 | Addressed (without qualifications) | Unspecified | Not addressed |
| 32 | Addressed (without qualifications) | All data and unused samples destroyed | Not addressed |
| 33 | Addressed (without qualifications) | Unspecified | Not addressed |
| 34 | Not addressed | Not addressed | Not addressed |
| 35 | Addressed (without qualifications) | Not addressed | Not addressed |
| 36 | Addressed (without qualifications) | Not addressed | Not addressed |
| 37 | Addressed (without qualifications) | Not addressed | Not addressed |
| 38 | Addressed (without qualifications) | Unspecified | Not addressed |
| 39 | Addressed (without qualifications) | Unspecified | Not addressed |
| 40 | Addressed (without qualifications) | Not addressed | Not addressed |
| 41 | Not addressed | Not addressed | Not addressed |
| 42 | Addressed (without qualifications) | All unused samples destroyed | Not addressed |
| 43 | Not addressed | Data collected up until declaration of withdrawal will not be removed | Not addressed |

**Existence of an Ability for the Child to Withdraw**

Child’s ability to withdraw addressed: 28/43
Not addressed: 15/43

**Extent of Child’s Ability to Withdraw**

All unused samples/data destroyed: 7/43
No further use of data/samples: 1/43
Data/samples collected until withdrawal maintained: 3/43
Unspecified: 9/43 %
Not addressed: 23/43

**Process for Handling Parental-Child Disagreement on Withdrawal**

Process for handling information/decisional entanglement: 0/43

Return of results issues.

| **Case Number** | **Return of Results** | **Process for Return** | **Who Returns/Contacts** |
| --- | --- | --- | --- |
| 11 | Not addressed | N/A | N/A |
| 33 | Not addressed | N/A | N/A |
| 38 | Not addressed | N/A | N/A |
| 1 | Not addressed | N/A | N/A |
| 2 | Not addressed | N/A | N/A |
| 4 | Not addressed | N/A | N/A |
| 12 | Not addressed | N/A | N/A |
| 13 | Not addressed | N/A | N/A |
| 15 | Not addressed | N/A | N/A |
| 16 | Not addressed | N/A | N/A |
| 21 | Not addressed | N/A | N/A |
| 28 | Not addressed | N/A | N/A |
| 34 | Not addressed | N/A | N/A |
| 35 | Not addressed | N/A | N/A |
| 39 | Not addressed | N/A | N/A |
| 40 | Not addressed | N/A | N/A |
| 3 | No return | N/A | N/A |
| 5 | No return | N/A | N/A |
| 6 | No return | N/A | N/A |
| 7 | No return | N/A | N/A |
| 8 | No return | N/A | N/A |
| 22 | No return | N/A | N/A |
| 14 | General/Aggregate | Subjects provided summary of results study conclusion | Investigators |
| 17 | General/Aggregate | - Mothers to receive summary of child’s developmental testing results - All participants to receive regular newsletters + summary of study | Investigators |
| 10 | General/Aggregate | Results available to parents after study | Investigators |
| 23 | General/Aggregate | Mothers to be kept informed of publications arising from the research | Investigators |
| 24 | General/Aggregate | Mothers to have access to general results and will be informed of publications arising from the research | Investigators |
| 36 | General/Aggregate | Study summary results provided to parents and daycare | Investigators |
| 37 | General/Aggregate | Study summary results provided to parents | Investigators |
| 26 | General/Aggregate + Individual (including incidental findings, with option) | - Participants have option to receive actionable incidental findings (not incl. paternity). - Participants have option to receive results of gene/gene mutation analysis - Participants have option to undergo further genetic testing and receive genetic counselling - Study summary results + clinical testing for family disorder provided upon study completion | Investigators (actionable incidental findings, individual results)  Geneticists/genetic counsellors (consultation for further genetic testing) |
| 27 | General/Aggregate + Individual (with option) (no incidental findings) | - Participants have option to receive results of gene/gene mutation analysis - Study summary results + clinical testing for family disorder provided upon study completion | Investigators |
| 41 | General/Aggregate + Individual (template includes possibility for incidental findings) | General research results available on website and/or another format | Investigators |
| 32 | Individual (including incidental findings) | Parents/child to be informed of incidental findings (not incl. paternity and adult onset disorders) + possible recommendation of repeat testing and discussion of risks/benefits | Investigators |
| 9 | Individual (including incidental findings, with option) | Parents and child have option to receive results of the study of family’s rare disease, as well as any other disease-causing variants | Investigators |
| 18 | Individual (including incidental findings, with option) | Parents and child (14-18 years of age) have option to receive news of actionable incidental findings (not incl. paternity) + genetic abnormality in sample + option to have news disclosed to referring physician + option to receive genetic counselling | Investigators  Geneticists/genetic counsellors (consultation for further genetic testing) |
| 20 | Individual (including incidental findings, with option) | Parents and child (14-18 years of age) have option to receive news of actionable incidental findings (not incl. paternity) + genetic abnormality in sample + option to have news disclosed to referring physician + option to receive genetic counselling | Investigators  Geneticists/genetic counsellors (consultation for further genetic testing) |
| 25 | Individual (with option) (no incidental findings) | Parents and child have option to receive individual genetic test results and new laboratory results that identify a specific genetic change in parent’s or child’s DNA sample | Investigators |
| 29 | Individual (with option) (no incidental findings) | Parents and child (14-18 years of age) have option to receive individual, study-related genetic mutation results after study completion | Investigators |
| 19 | Individual (including incidental findings) + General/Aggregate (with option) | - Parents may obtain information concerning study progress or general results. - Identification of actionable genetic variations to be disclosed (no paternity disclosure) - Option to have independent clinic explain results and confirm via new blood sample - Option to have genetic counselling | Investigators  Independent genetic clinic (to explain results and confirm test results via new blood sample)  Genetic counsellor (to discuss test results) |
| 30 | Individual (including incidental findings) + General/Aggregate (with option) | - Parents may obtain information concerning study progress or general results. - Identification of actionable genetic variations to be disclosed (no paternity disclosure) - Option to have independent clinic explain results and confirm via new blood sample - Option to have genetic counselling | Investigators  Independent genetic clinic (to explain results and confirm test results via new blood sample)  Genetic counsellor (to discuss test results) |
| 31 | Individual (with option) (no incidental findings) + General/Aggregate (with option) | Participants given option to receive individual and general research results, with disclosure of method for dissemination | Investigators |
| 42 | Individual (only for tumour biopsy) (including incidental findings)  No return for biobank/future research specimens | - If tumour biopsy sample is reviewed by expert pathologists, results will be given to patient’s doctor - Incidental findings to be relayed by researchers to patient’s doctor | Doctor |
| 43 | Not addressed | N/A | N/A |

**Return of Results**

**Not addressed: 17/43 No return: 6/43
General/Aggregate: 7/43 Individual: 7/43 General/Aggregate + Individual: 6/43**Privacy and confidentiality issues.

| **Case Number** | **Parents’ Right to  Access Information Concerning their Child** | **Nomenclature for Data and/or Sample Identifiability** |
| --- | --- | --- |
| 1 | Not addressed | Unspecified |
| 2 | Not addressed | Unspecified |
| 3 | Not addressed | Coded |
| 4 | Not addressed | Anonymised |
| 5 | Not addressed | Coded samples and unspecified for data |
| 6 | Not addressed | Coded samples and unspecified for data |
| 7 | Not addressed | Coded samples and unspecified for data |
| 8 | Not addressed | Coded samples and unspecified for data |
| 9 | Not addressed | Coded samples and anonymized data |
| 10 | Not addressed | Unspecified |
| 11 | Not addressed | Unspecified |
| 12 | Not addressed | Unspecified |
| 13 | Not addressed | Unspecified |
| 14 | Not addressed | Unspecified |
| 15 | Not addressed | Coded |
| 16 | Not addressed | Unspecified |
| 17 | Not addressed | Coded samples and unspecified for data |
| 18 | Not addressed | Coded samples and unspecified for data |
| 19 | Not addressed | Coded samples and unspecified for data |
| 20 | Not addressed | Coded samples and unspecified for data |
| 21 | Not addressed | Coded |
| 22 | Not addressed | Coded |
| 23 | Not addressed | Coded |
| 24 | Not addressed | Coded |
| 25 | Not addressed | Anonymised |
| 26 | Not addressed | Anonymised samples and coded data |
| 27 | Not addressed | Anonymised samples and coded data |
| 28 | Not addressed | Unspecified |
| 29 | Not addressed | Coded |
| 30 | Not addressed | Coded |
| 31 | Not addressed | Unspecified |
| 32 | Not addressed | Coded |
| 33 | Not addressed | Unspecified |
| 34 | Not addressed | Coded |
| 35 | Not addressed | Unspecified |
| 36 | Not addressed | Coded |
| 37 | Not addressed | Unspecified |
| 38 | Not addressed | Unspecified |
| 39 | Not addressed | Coded |
| 40 | Not addressed | Coded |
| 41 | Not addressed | Coded |
| 42 | Not addressed | Coded |
| 43 | Not addressed | Coded |

**Parents’ Right to Access Information Concerning Their Child**

Not addressed: 0/43

**Nomenclature for Data and/or Sample Identifiability**

Unspecified: 14/43
Coded: 16/43
Coded samples and unspecified for data: 9/43
Anonymised data and/or samples: 4/43

**Retention and transfer of the child’s data and/or samples**.

| **Case Number** | **Retention Period(s) of**  **Samples/Data** | **Transfer of Samples/Data to  Another Location** |
| --- | --- | --- |
| 1 | Retention of data/samples (w/periods) | Not addressed |
| 2 | Not addressed | Not addressed |
| 3 | Retention of samples (indefinite) | Disclosure that data/samples may be transferred to another location |
| 4 | Not addressed | Not addressed |
| 5 | Retention of samples (up to 25 years) | Disclosure that samples may be transferred to another location |
| 6 | Retention of samples (up to 25 years) | Disclosure that samples may be transferred to another location |
| 7 | Retention of samples (up to 25 years) | Disclosure that samples may be transferred to another location |
| 8 | Retention of samples (up to 25 years) | Disclosure that samples may be transferred to another location |
| 9 | Retention of data/samples (no period) | Disclosure that data/samples may be transferred to another location |
| 10 | Retention of data (no period) | Not addressed |
| 11 | Retention of data/samples (w/periods) | Disclosure and discussion of procedure for transfer of data/samples to another location |
| 12 | Not addressed | Not addressed |
| 13 | Retention of data/samples (w/periods) | Not addressed |
| 14 | Not addressed | Not addressed |
| 15 | Retention of samples (10 years)  Retention of data (20 years) | Disclosure and discussion of procedure for transfer of samples to another location |
| 16 | Retention of data (until child reaches age of majority or re-consents) | Disclosure and discussion of procedure for transfer of data to another location |
| 17 | Retention of samples (indefinite)  Retention of data (12 years even if withdrawal; otherwise indefinite) | Disclosure that data/samples may be transferred to another location |
| 18 | Retention of samples (until study complete)  Retention of data (no period) | No transfer of data/samples |
| 19 | Retention of samples (indefinite) | Not addressed |
| 20 | Retention of samples (until study complete)  Retention of data (no period) | No transfer of data/samples |
| 21 | Retention of samples (indefinite)  Retention of data (no period) | Not addressed |
| 22 | Retention of samples (indefinite)  Retention of data (25 years even if withdrawal; otherwise indefinite) | Disclosure that data/samples may be transferred to another location |
| 23 | Retention of samples (indefinite)  Retention of data (7 years even if withdrawal; otherwise indefinite) | Disclosure that data/samples may be transferred to another location |
| 24 | Retention of samples (20 years even if withdrawal; otherwise indefinite)  Retention of data (25 years even if withdrawal; otherwise indefinite) | Disclosure that data/samples may be transferred to another location |
| 25 | Retention of samples (until study complete or indefinite, at option of participant) | Disclosure that samples may be transferred to another location |
| 26 | Not addressed | Disclosure that data/samples may be transferred to another location |
| 27 | Not addressed | Disclosure that data/samples may be transferred to another location |
| 28 | Retention of data/samples (w/periods) | Disclosure that data may be transferred to another location |
| 29 | Retention of data/samples (10 years) | Not addressed |
| 30 | Retention of samples (indefinite) | Not addressed |
| 31 | Retention of data/samples (w/periods) | Not addressed |
| 32 | Retention of samples (indefinite)  Retention of data (until study project complete) | Disclosure and discussion of procedure for transfer of data/samples to another location |
| 33 | Retention of data/samples (w/periods) | Not addressed |
| 34 | Not addressed | Not addressed |
| 35 | Retention of paper data (3 years) and electronic data (indefinite) | Not addressed |
| 36 | Retention of data (minimum 5 years) | Not addressed |
| 37 | Not addressed | Not addressed |
| 38 | Retention of data/samples (w/periods) | Not addressed |
| 39 | Retention of data (25 years) | Not addressed |
| 40 | Not addressed | Not addressed |
| 41 | Retention of data/samples (w/periods) | Disclosure that data/samples may be transferred to another location |
| 42 | Retention of data/samples (indefinite – if participant agrees to biobanking option) | Disclosure that data/samples may be transferred to another location |
| 43 | Retention of samples (15 years) (or indefinite if participant agrees to biobanking option)  Retention of data (25 years) | Disclosure that data/samples may be transferred to another location |

**Retention Period(s) of Samples/Data**

Not addressed: 9/43
Indefinite: 12/43
Time periods: 18/43
Indefinite + Time periods: 4/43

**Transfer of Samples/Data to another Location**

Not addressed: 20/43
No transfer: 2/43
Disclosure of transfer: 17/43
Disclosure of transfer + procedure: 4/43
